# Revelation of comprehensive cell profiling of primary and metastatic tumour ecosystems in oral squamous cell carcinoma by single-cell transcriptomic analysis

**DOI:** 10.7150/ijms.97404

**Published:** 2024-08-26

**Authors:** Yin-han Liao, Li Chen, Bing-hua Feng, Wei Lv, Xuan-ping Huang, Hao Li, Cui-ping Li

**Affiliations:** 1 College & Hospital of Stomatology, Guangxi Medical University, Nanning 530021, P. R. China.; 2 Guangxi Key Laboratory of the Rehabilitation and Reconstruction of Oral and Maxillofacial Research, Nanning 530021, P. R. China.; 3 Guangxi Health Commission Key laboratory of prevention and treatment for oral infectious diseases, Nanning 530021, P. R. China.; 4 Guangxi Clinical Research Center for Craniofacial Deformity, Nanning 530021, P. R. China.

**Keywords:** single-cell sequencing, oncogene, oral squamous cell carcinoma, metastasis

## Abstract

**Background:** The analysis of single-cell transcriptome profiling of tumour tissue isolates helps to identify heterogeneous tumour cells, neighbouring stromal cells and immune cells. Local metastasis of lymph nodes is the most dominant and influential biological behaviors of oral squamous cell carcinoma (OSCC) in terms of treatment prognosis. Understanding metastasis initiation and progression is important for the discovery of new treatments for OSCC and prediction of clinical responses to immunotherapy. However, the identity of metastasis-initiating cells in human OSCC remains elusive, and whether metastases are hierarchically organized is unknown. Therefore, this study was conducted to understand the cellular origins and gene expression signature of OSCC at the single-cell level.

**Methods:** Single-cell RNA sequencing (scRNA-seq) was used to analyze cells from tissue of para-carcinoma (PCA: adjacent normal tissue not less than 2 cm from the tumour), carcinoma (CA), lymph node metastasis (LNM) from patients with OSCC and PCA and CA tissue from patients with second primary OSCC (SPOSCC) after radiotherapy of nasopharyngeal carcinoma (NPC). The cell types and their underlying functions were classified. The comparisons were then conducted between the homology and heterogeneity from cell types and both conservative and heterogeneous aspects of evolution were identified. Immunohistochemistry was performed to verify the makers of cell clusters and the expression level of novel genes.

**Results:** A single-cell transcriptomic map of OSCC was created, including 16 clusters of PCA cells, 17 clusters of CA cells, 14 clusters of left LNM cells, and 14 clusters of right LNM cells. We also discovered two novel types of cells including CD1C-CD141-dendritic cells and CD1C+_B dendritic cells. Most of the non-cancer cells are immune cells, with two distinct clusters of T lymphocytes, B lymphocytes, CD1C-CD141-dendritic cells+ and CD1C+_B dendritic cells. We also classified cells into 15 clusters for SPOSCC after radiotherapy of NPC. Determining the upregulated expression levels of IL1RN and C15orf48 as novel markers using immunohistochemistry facilitated the correct classification of OSCC including SPOSCC after radiotherapy of NPC and the prediction of their prognosis.

**Conclusions:** The findings provided an unprecedented and valuable view of the functional states and heterogeneity of cell populations in LNM of OSCC and SPOSCC after radiotherapy of NPC at single-cell genomic resolution. Moreover, this transcriptomic map discovered new cell types in mouth, and novel tumour cell-specific markers/oncogene.

## Introduction

Some studies about genome and transcriptome have fragmented the disease into the different subtypes of disease, and showed aberrant regulatory programs, and mutations of driving for many cancers^1^, ^2^. However, these researches were only dependent on profiling technologies which could analyze the underlying causes of cancers but the ability to capture intra-tumoral heterogeneity was limited. A lot of evidence showed that it was critical to explain diverse aspects of tumour biology such as stratification of immune cells in the tumour microenvironment (TME), heterogeneity within the tumour, interactions among malignant and non-malignant cells within the TME and so on^1^, ^3^ . Tumour heterogeneity manifests itself as differences between tumours in which different stages, genetic lesions or expression programs are associated with different outcomes or therapeutic responses, posing a major challenge to the diagnosis, treatment and prognosis of cancer^4^ . In addition, cells from the same tumour may contain different mutations or exhibit different phenotypic or epigenetic states. This intra-tumour heterogeneity is increasingly recognized as a determinant of treatment failure and disease recurrence^5^, ^6^.

For care of refractory cancer cases, patients tend to accept non-invasive serial and sensitive monitoring of circulating rare recurrent tumour cell populations, and assessment of tumour heterogeneity^7^. The single-cell sequencing is a newly developed technology, which is a promising systematic and comprehensive method to expose genetic alteration within tumours, rebuild tumour cloning structures, and authenticate common mutant genes situated in the tumour subgroup^8^. With the rapid evolution of microfluidics technology, single-cell RNA-sequencing (scRNA-seq) has been applied to enable synchronous analysis of thousands of cells taken from living tissue samples at the single-cell level^9^. Up to now, scRNA-seq analysis had been applied in cancerous and immune cells from some cancer patients, which displayed the exhaustion signature of a typical T cell and its relationship with T cell activation^10^, and beyond that, infiltration analysis of lymphocytes at the single-cell level had been given a detailed explanation of the role in these cells in a highly complex TME. Single-cell genome analysis is expected to have utility in treating cancer in clinical^11^. Better molecular targets for prognosis and treatment were identified by understanding the level of transcriptome heterogeneity in the tumour entity and precisely characterizing the gene expression of tumour and microenvironment^4^.

Head and neck cancer ranks sixth among malignant tumours and oral cancer has 28% of head and neck cancer. Oral squamous cell carcinoma (OSCC) ranks first among oral cancers, accounting for more than 90%, with more than 350,000 new cases and 170,000 deaths worldwide each year^12^, ^13^. The tendency to metastasize for OSCC varies with the variation of the grading of pathological histology, and the tendency to metastasize becomes higher from well-differentiated keratinizing to undifferentiated non-keratinizing carcinoma^14^. The development of OSCC is closely associated with dysregulation of protooncogenes/oncogenes. In the development of OSCC, most OSCC have a clear stage of precancerous damage, going through a process from normal to precancerous lesions, carcinoma in situ, and invasive carcinoma. Some previous studies have provided an in-depth insight into the etiology and pathogenesis of OSCC^15^, ^16^, but there are still challenges in understanding the interindividual differences in the pathogenesis at the molecular level and determining whether changes in key gene expression may be participated in the onset, progression, or accommodate of reactions or resistance to antitumor medicines. Therefore, high-throughput single-cell sequencing of multiple OSCC specimens revealed the cloning and genetic complexity of tumours.

The ecosystem of cells in OSCC is tremendously complex with various phenotypes, genotypes and epigenetic states, and its development processes are multi-perspective, multi-level and adjusted by the levels of DNA, transcription, post-transcription and protein. Moreover, scRNA-seq is an emerging technology that can be applied to understanding the TME of OSCC at a high resolution^17^-^19^. Given that scRNA-seq for lymph node metastasis (LNM) from OSCC and second primary OSCC (SPOSCC) after radiotherapy of nasopharyngeal carcinoma (NPC) has not been reported thus far. In this study, single cells were isolated from carcinoma (CA) tissue and para-carcinoma (PCA: adjacent normal tissue not less than 2 cm from the tumour) and tissue samples in LNM and then scRNA-seq was used to profile cell populations from primary and secondary OSCC clumps; moreover, single cells were also isolated from CA and PCA tissue samples of SPOSCC after radiotherapy of NPC. To verify the accuracy of cell populations, we performed immunostaining of some markers for individual cell populations using in the collected clinical cases and then verified the expression of novel genes. We identified two new genes, which mutate in some cell populations of CA level group at low frequencies, but in PCA group at high frequencies. Moreover, this study can find these cells which have inherent variability in their expression of multiple transcriptional procedures associated with tumour metastasis. To further improve clinical practice and patient outcomes, we need to strengthen the study of molecular biology in the underlying pathogenesis of disease to and understand the level of transcriptomic heterogeneity in tumour entities, so that new beneficial molecular markers can be proposed and applied to treatment.

## Materials and methods

### Tissue specimens

In this study, three patients with pathologically diagnosed OSCC and two with SPOSCC after radiotherapy of NPC were used for scRNA-seq analysis. Fresh tissue of tongue was acquired from the OSCC patients and washed with an icy saline solution for subsequent lymphocyte isolation. All tissue samples were at least 2 cm in size. This study had acquired informed consent from the subjects of study and was approved by the Medical Ethics Committee of the affiliated hospital of Guangxi Medical University (No: 2022068).

### Tissue acquisition and preparation of single cell suspension

Fresh OSCC tumour tissue, paired PCA (adjacent normal tissue not less than 2 cm from the tumour) and LNM tissue was obtained from the patients in the operating room. The distance between the adjacent normal tissue and the tumour tissue was not less than 2 cm. Fresh sample tissue were placed into solution (precooled HBSS and 1% penicillin-streptomycin) immediately. Immediately afterwards, they were rinsed three times with 4℃ DPBS on the ultra-clean table. Samples were trimmed with tissue scissors to 1-2mm in diameter, washed three times again with DPBS and then added to 0.25% trypsin solution and digested in the incubation phase at 37℃ for 40 min, observed every 5 min. Digestion was terminated by adding DMEM complete medium containing 10% FBS, and the suspension was filtered twice through a 70μm mesh nylon net and then centrifuged at 1000 x g for 10 min. The supernatant was discarded and 2 mL of DMEM complete medium was added to collect the dissociated single cells. Next, 3 mL of 1x RBC lysis buffer was added and after 5 min, the cells were centrifuged at 300 x g for 5 min to remove the red blood cells, and the supernatant was discarded and the cells were resuspended in complete medium. After obtaining the single-cell suspension, the live cells were detected with a hemocytometer using trypan blue.

### Samples were processed by 10 x Genomics and cDNA library preparation

After multiple fresh tissue samples were obtained, and digested into individual cells, the single-cell suspension was successfully prepared and then treated by applying 10 x Genomics Chromium Single Cell 3'Reagents Kit Version 2 (https://support.10xgenomics.com/single-cellgene-expression/index/doc/user-guide-chromium-singlecell-3-reagent-kits-user-guide-v2-chemistry). Single-cell suspension was filtered by applying a 40-mm cell filter, and then a hemocytometer with trypan blue was used to calculate the proportions of viable cells. Subsequently, each sample with 10,000 cells in total were separated and recovered. Single cells, gel beads and oils were added into the 10 x Genomics Single Cell A Chip following the 10 x specification and then the machine started to run. After the water droplets were formed, samples were transported and then reverse-transcribed into cDNA, which were operated on a specific machine (Bio-Rad). CDNAs were repaired by using Recovery Agent from 10 x Genomics and then washed by using a Silane Dyna Bead following the instructions from the manual. Purification cDNA was amplified and then conducted for ten cycles, which was cleaned by specific selection beads. A Qubit2.0 Fluorometer (Invitrogen) examined the cDNA concentration of all samples. The cDNA libraries were created according to the directions of Chromium Single Cell 3' Reagent Kits version 2.

### Preliminary sequencing results from scRNA-Seq

A Hiseq Xten (Illumina, San Diego, CA) was used to sequence samples and the run parameters for sequencing based on the following parameters: read 1: 150 cycles, read 2: 150 cycles, index: 14 cycles. FASTQ files with Cell Ranger were acquired by converting preliminary sequencing results (bcl files), which were subsequently brought into a common alignment with the human genome reference sequence (GRCh38). Cell Ranger was also applied which was in agreement with raw data analysis and a file was subsequently generated which came from a lot of comprise including a barcodes table, a genes table, and a gene expression matrix. Ultimately, an overall website was acquired which consisted of the amount of cells, median number of genes tested, the saturation and depth of sequencing.

### Bioinformatics analysis

TopGO software was used to determine the gene ontology (GO) feature of the clusters. Genes were used for enrichment, an average logFC value of which was above zero and p value adjusted was less than 0.05. The “compareCluster” function of on these selective genes was conducted depending on the P-value node (0.05) to compare the enrichments between different clusters.

### Immunohistochemistry verification

A total of 51 PCA, 41 CA, 67 LNM, and 11 SPOSCC after radiotherapy of NPC were used for cell clusters verification by immunohistochemistry. Samples used for IHC validation of selected markers were located in the lateral margins of the tongue as well as in the metastatic lymph nodes. There were no statistically significant differences in age, gender, tumour stage, tumour size, tumour differentiation degree, smoking state and drinking state among these groups (P > 0.05). The available clinical characteristics were summarized in Table 1. The samples were retrospective.

Each excised tissue was sized as a 5mm x 4mm x 3mm block and then fixed in 4% paraformaldehyde for 24-48 h. These tissue for paraffin embedding were obtained from patients undergoing OSCC, carcinoma in situ, OSCC after radiotherapy of NPC, and enucleated cell tumour resection. Sections were cut to a thickness of 3μm, after which immunohistochemical staining was performed. Tissue was blocked in goat serum in PBS for 15 min at room temperature. The slides were incubated with anti-CD3 antibody (rabbit anti-human/mouse, 1:100, ab135372; Abcam), anti-CD20 antibody (rabbit anti-human/monkey, 1:50, ab78237; Abcam), anti-CD79A antibody (mouse anti-mouse/rat/human, 1:100, sc-20064; Santa), anti-S100A2 antibody (rabbit anti-human, 1:500, ab109494; Abcam), anti-KRT17 antibody (rabbit anti-mouse/rat/human, 1:600, 12434S; CST), anti-C15orf48 antibody (rabbit anti-human, 1:200, HPA012943; Sigma), anti-IL1RN antibody (rabbit anti-mouse/rat/human, 1:400, ab124962; Abcam), and PBS control prepared in blocking solution at 4°C overnight. After washing in PBS, the tissue was incubated with secondary antibodies (ZB-2305; Zsbio) for 15 min at room temperature. Finally, we used DAB (ZLI-9018; Zsbio) for staining and hematoxylin for nucleation.

The diagnostic results were interpreted by two or more senior pathologists in a “double-blind” manner, with five randomly selected fields of view for each section. The intensity of positivity had been also assessed in the samples by the following principles: each sample was scored by multiplying degree of staining (0: negative coloring; 1: light yellow; 2: light brown; 3: dark brown) and range of positivity (1: 0-25%; 2: 26-50%; 3: 51-75%; 4: 76-100%) under a light microscope and the score was ≥ 4, which was considered as positive.

### Clinicopathologic data analysis and statistical analysis

This study obtained the basic characteristics and relevant clinical details from clinical examination of the research objects. The correlation between different clinicopathologic factors and the protein expression from immunohistochemistry verification results was analyzed by using Chi-square test, McNemar's test and Fisher's exact tests from SPSS 22.0 software package. The basic characteristics and clinicopathologic factors included the following information: (1) two grades of age brackets (≤ 50 years/> 50 years); (2) gender (male/female); (3) tumour stage (I/II stage/III/IV stage); (4) tumour size (< 3cm/≥ 3cm); (5) tumour differentiation degree (highly differentiated squamous cell carcinoma/lowly differentiated squamous cell carcinoma); (6) smoking state (smoking/non-smoking); (7) drinking state (drinking/non-drinking).

## Results

### ScRNA-Seq profiling and unbiased clustering of cells between PCA, CA, and LNM with OSCC

A total of 11,311 cells were isolated and sequenced in CA, mean reads per cell were 25,725 reads and the median genes per cell were 1502 detectable genes; a total of 23,664 genes were detected and the median UMI counts per cell was 5032 in CA group.

**Figure 1:**
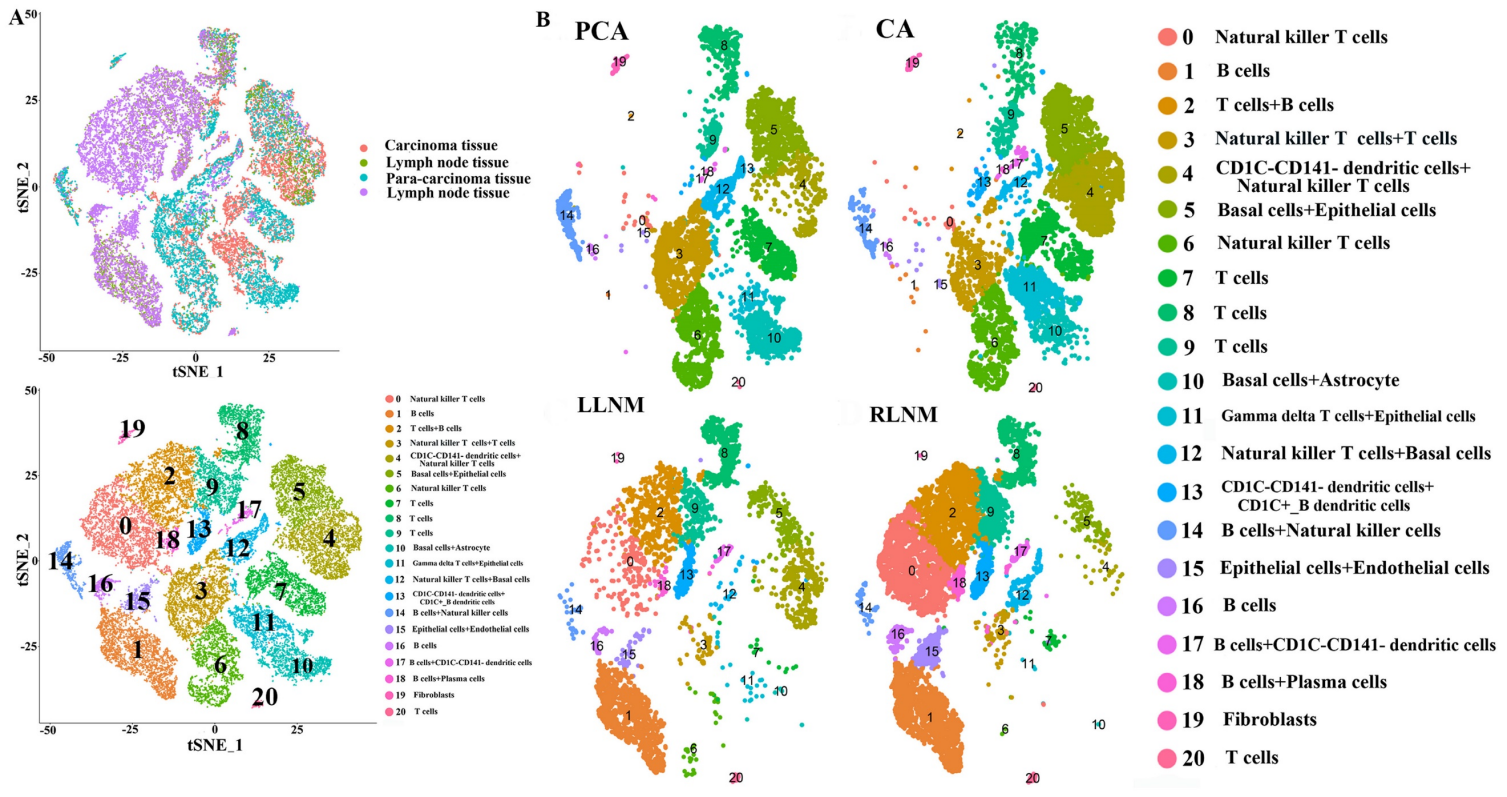
High-throughput 10× scRNA-seq reveals the cell populations of oral squamous cell carcinoma. (A) Overview of the scRNA-seq approach using tissue samples from para-carcinoma (PCA), carcinoma (CA), left lymph node metastasis (LLNM), right lymph node metastasis (RLNM) from patients of oral squamous cell carcinoma that were processed into a single-cell suspension, and scRNA-seq analysis using the 10× platform. (B) t-SNE plot representation of the distribution of PCA, CA, LLNM and RLNM and clusters are colored and distinctively labeled.

A total of 11,028 cells were isolated and sequenced in PCA, mean reads per cell were 29,004 reads and the median genes per cell were 2045 detectable genes; a total of 23,295 genes were detected and the median UMI counts per cell was 7477 in PCA group. A total of 10,427 cells were isolated and sequenced in right LNM tissue, mean reads per cell were 29,494 reads and the median genes per cell were 1094 detectable genes; a total of 21,042 genes were detected and the median UMI counts per cell was 3792 in right LNM group. A total of 4,833 cells were isolated and sequenced in left LNM tissue, mean reads per cell were 59,778 reads and the median genes per cell were 1201 detectable genes; a total of 23,226 genes were detected and the median UMI counts per cell was 4402 in left LNM group. Under stringent quality controls by seurat17 (https://satijalab.org/seurat/), a total of 21 distinct cell clusters were identified in clustering analysis. We classified cells into cluster 0-20 on the basis of the marker genes of each type of cells, thereby corresponding to natural killer T (NKT) cells (0), B cells (1), T cells+B cells (2), NKT cells+T cells (3), CD1C-CD141-dendritic cells+NKT cells (4), basal cells+epithelial cells (5), NKT cells (6), T cells (7), T cells (8), T cells (9), basal cells+astrocytes (10), gamma delta T cells+epithelial cells (11), NKT cells+basal cells (12), CD1C-CD141-dendritic cells+CD1C+_B dendritic cells (13), B cells+NKT cells (14), epithelial cells+endothelial cells (15), B cells (16), B cells+CD1C-CD141-dendritic cells (17), B cells+plasma cells (18), fibroblasts (19), and T cells (20), respectively (Figure 1). Some differences in cell homogeneity were found between different comparison groups.

### The cell types in each cluster and the marker genes identified in OSCC

**Figure 2:**
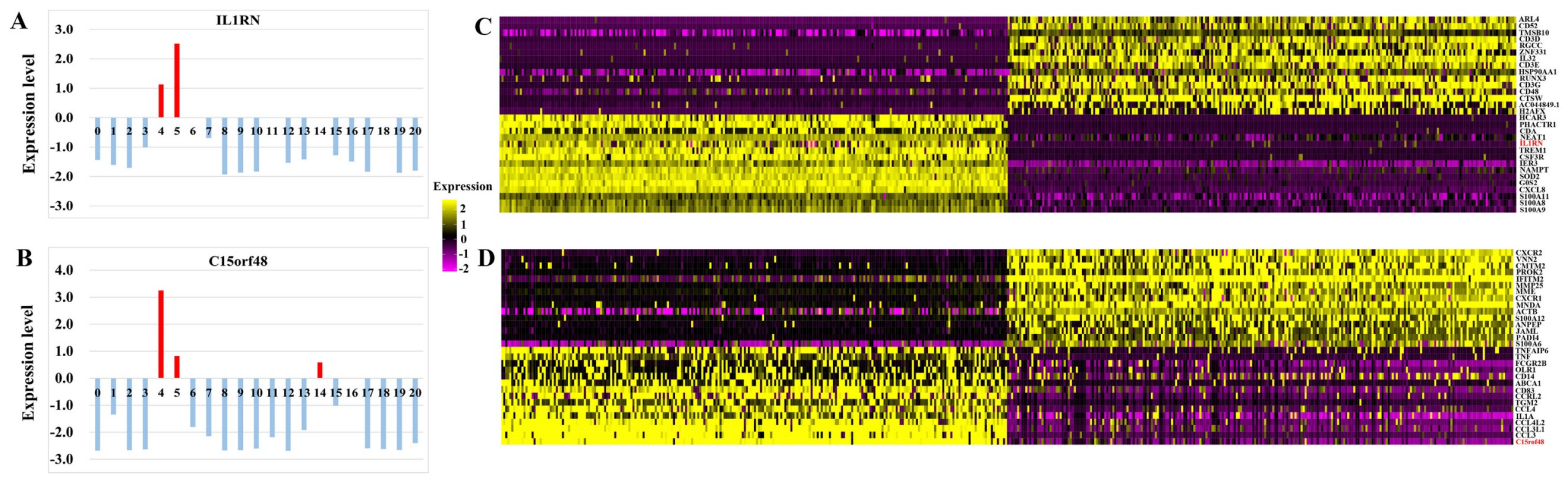
(A) The expression levels of IL1RN in each cell clusters. (B) The expression levels of C15orf48 in each cell clusters. (C) Heat map showing the marker genes including IL1RN in a cell cluster. (D) Heat map showing the marker genes including C15orf48 in a cell cluster.

(1) Some differentially-expressed cell clusters can be found in CA groups when compared with PCA. The number of cells of cluster 4, 11 in CA group was significantly higher than those in PCA group and the number of cells of cluster 3, 10, 12 in PCA group was significantly higher than those in CA group (Table 2; Figure 1). Some genes including IL1RN and C15orf48 were highly expressed significantly in cluster 4 and 5 compared with those of other clusters, and they were also highly expressed significantly in CA group compared with PCA group (Table 2; Figure 2). S100A2 as a maker of basal cells was also highly expressed significantly in CA group compared with PCA group (Table 2).

(2) Some differentially-expressed cell clusters and genes can be found in LNM group when compared with CA and PCA. For example, the number of cells of cluster 0, 1, 2, 15,16 in LNM group was significantly higher than those in CA and PCA groups and the number of cells of cluster 3, 4, 5, 6, 7, 10, 11, 14 in LNM group was significantly lower than those in CA and PCA groups (Figure 1). Some genes including IL1RN, C15orf48 were highly expressed significantly in cluster 4 and 5 compared with those of other clusters, which were highly expressed significantly in CA group compared with PCA, LNM group (Table 2; Figure 2). S100A2 was also highly expressed significantly in CA group compared with PCA group (Table 2). Some genes including CD20, CD79A as makers of B cells in cluster 1, 2, 16 were highly expressed significantly in LNM group compared with those of CA, PCA groups (Table 2; Figure 2). There were no significant differences for CD 3 as makers of T cells in cluster 2, 7, 8, 9, 20 in LNM group compared with those of CA, PCA groups (Table 2; Figure 1). CD 31 as makers of endothelial cells were highly expressed significantly in LNM group compared with those of CA, PCA groups (Table 2; Figure 1). Cytokeratin 5 as makers of keratinocytes were highly expressed significantly in CA, PCA groups compared with those of LNM group (Table 2; Figure 1). There were no significant differences for CD 66 as makers of neutrophils in LNM group compared with those of CA, PCA groups (Table 2; Figure 1).

### ScRNA-Seq profiling and unbiased clustering of cells between PCA, CA, LNM from patients with OSCC and PCA and CA tissue from SPOSCC patients after radiotherapy of NPC

**Figure 3:**
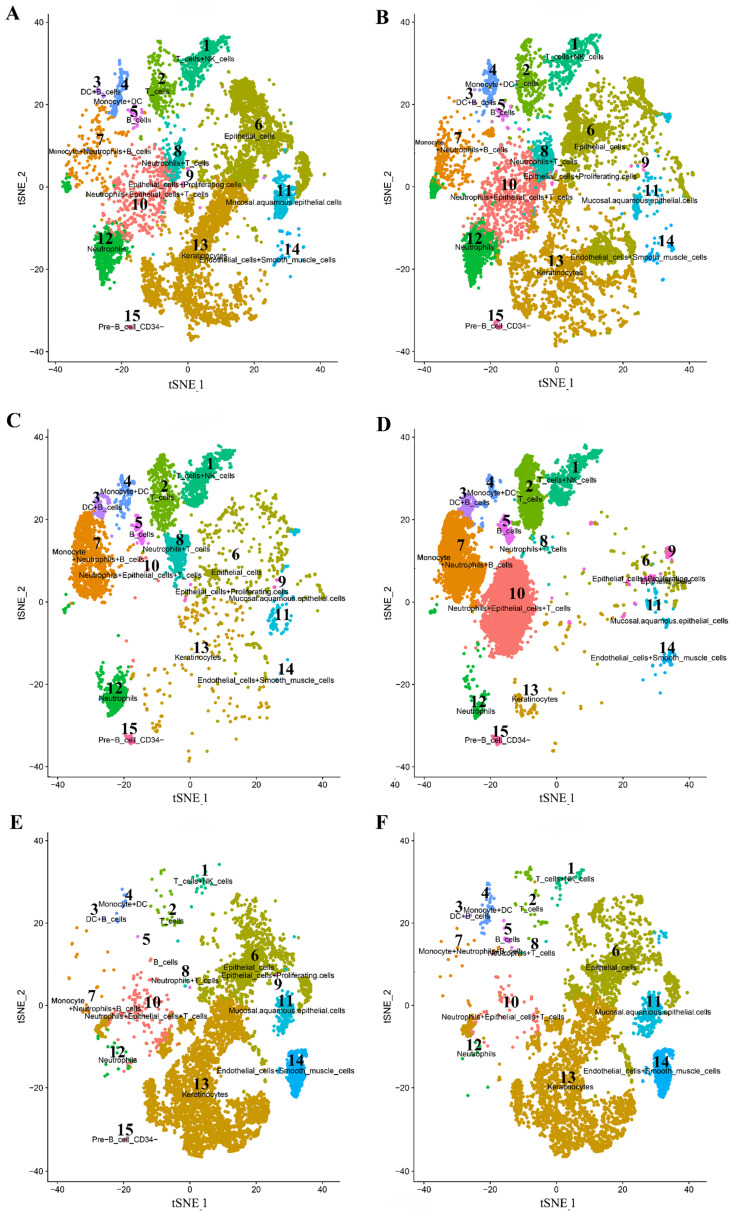
High-throughput 10 × scRNA-seq reveals the cell populations of second primary OSCC (SPOSCC) after radiotherapy of nasopharyngeal carcinoma (NPC). (A) t-SNE plot representation from para-carcinoma (PCA) tissue and clusters are colored and distinctively labeled. (B) t-SNE plot representation from carcinoma (CA) tissue and clusters are colored and distinctively labeled. (C) t-SNE plot representation from left lymph node metastasis (LLNM) tissue and clusters are colored and distinctively labeled. (D) t-SNE plot representation from right lymph node metastasis (RLNM) tissue and clusters are colored and distinctively labeled. (E) t-SNE plot representation from PCA tissue from SPOSCC after radiotherapy of NPC and clusters are colored and distinctively labeled. (F) t-SNE plot representation from CA tissue from SPOSCC after radiotherapy of NPC and clusters are colored and distinctively labeled.

A total of 15 distinct cell clusters were identified in clustering analysis. We classified cells into 15 clusters for SPOSCC patients after radiotherapy of NPC on the basis of the marker genes of each type of cells, thereby corresponding to T cells+NK cells (1), T cells (2), DC+B cells (3), monocyte+DC cells (4), B cells (5), epithelial cells (6), monocyte+neutrophils+B cells (7), neutrophils+T cells (8), epithelial cells+proliferating cells (9), neutrophils+epithelial cells+T cells (10), mucosal.aquamous.epithelial.cells (11), neutrophils (12), keratinocytes (13), endothelial cells+smooth muscle cells (14), pre B cells CD34- (15), respectively (Figure 3). Some differences in cell homogeneity were found between different comparison groups. Interestingly, the marker genes were identified in each cluster and we defined the cell type of each cluster.

### The cell type of each cluster and the marker genes in each cluster identified in SPOSCC patients after radiotherapy of NPC

Some differentially-expressed cell clusters can be found in SPOSCC patients after radiotherapy of NPC when compared with CA, PCA, LNM groups. For example, the number of cells of cluster 1, 2, 5, 7, 8, 12 in SPOSCC patients after radiotherapy of NPC group was significantly lower than those in CA, PCA, LNM groups, and the number of cells of cluster 13, 14 in SPOSCC patients after radiotherapy of NPC group was significantly lower than those in CA, PCA, LNM groups (Figure 3).

**Figure 4:**
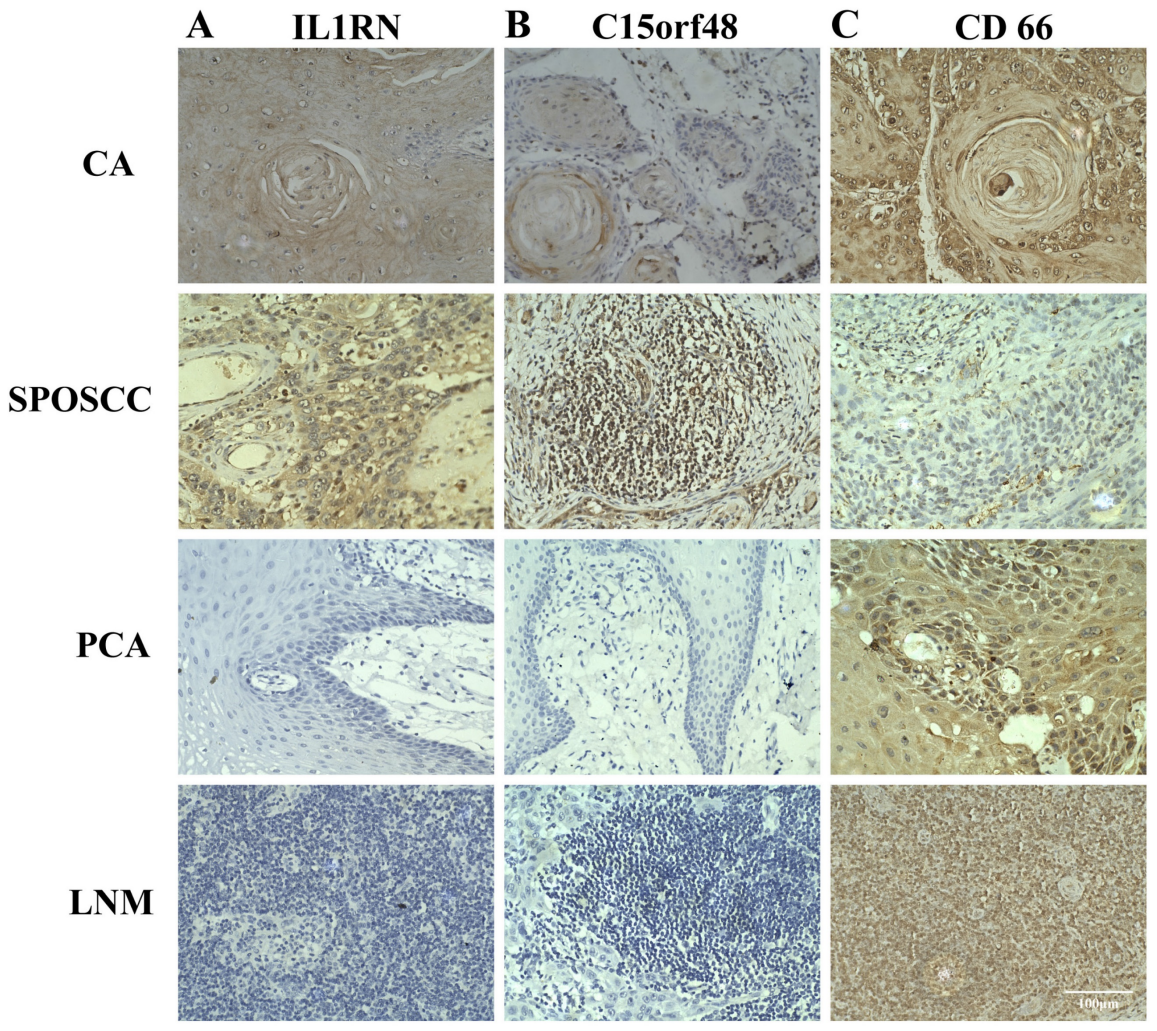
The expression of IL1RN, C15orf48, CD66 in individual samples assayed by IHC. A, B and C represented the expression of IL1RN, C15orf48, CD66 in cancer tissues (CA), the corresponding paracancerous tissues (PCA), lymph node metastasis (LNM), second primary OSCC (SPOSCC) after radiotherapy of nasopharyngeal carcinoma (NPC) and at least five fields per tissue (scale bar:100μm), respectively.

The number of cells of cluster 3, 6, 7 in CA, PCA, SPOSCC patients after radiotherapy of NPC groups was significantly lower than those in LNM group, and the number of cells of cluster 13 in CA, PCA, SPOSCC patients after radiotherapy of NPC groups was significantly lower than those in LNM group (Figure 3). The novel gene-IL1RN was highly expressed significantly in SPOSCC patients after radiotherapy of NPC group compared with other groups, IL1RN, C15orf48 were highly expressed significantly in CA, SPOSCC patients after radiotherapy of NPC groups compared with PCA, LNM groups (Table 3; Figure 4). CD 66 as a maker of neutrophils was also lowly expressed significantly in SPOSCC patients after radiotherapy of NPC group compared with other groups (Table 3; Figure 4), but there were no significant differences for CD 66 between LNM group and those of CA, PCA groups (Table 2; Figure 3). Cytokeratin 5 as makers of keratinocytes was highly expressed significantly in CA, PCA, SPOSCC patients after radiotherapy of NPC groups compared with those of LNM group (Table 3). CD20, CD79A as makers of B cells were highly expressed significantly in LNM group compared with those of CA, PCA and SPOSCC patients after radiotherapy of NPC groups (Table 2; Figure 3). There were no significant differences for CD 3 as makers of T cells in all groups (Table 3). Some genes including CD 31 as makers of endothelial cells were highly expressed significantly in SPOSCC patients after radiotherapy of NPC, LNM groups compared with those of CA, PCA groups (Table 3).

### Clinicopathologic findings of patients with OSCC

When in comparisons with all the clinicopathologic parameters, CD20 was significantly higher in OSCC patients with ≤ 50 years/I/II stage/smoking than that in patients with > 50 years/III/IV stage/no smoking (Table 4). CD79 was significantly higher in low differentiated squamous-cell carcinoma patients than in well differentiated squamous cell carcinoma patients (Table 4). CD31 was significantly higher in OSCC patients with smoking and drinking than in patients without smoking and drinking (Table 4). In terms of all validated makers expression, there was no statistically significant difference in any other comparison groups (Table 4).

## Discussion

The function of the immune system in the progression of the tumour becomes a heat point of present scientists” concern, and the findings of these researches have supplied some coordinate evidence for successful immunotherapy in some kinds of cancers^20^, ^21^, OSCC was no exception^14^. This transcriptome data provides a comprehensive resource for understanding multidimensionally the constituents and characterizations of cells, which is useful for identifying developmental hierarchies, and patterns of the immune infiltration connected with the occurrence and development, prevention, diagnosis, and therapy of tumour. In this study, we provide a means of characterizing primary OSCC tumours and corresponding LN metastases, OSCC caused by radiotherapy for NPC. Our study identifies 21 large subsets and unique subpopulations with high quantity and quality of single-cell data and cell populations were also authenticated based on known surface marker expression, which allowed us to map a complex cellular ecosystem and developmental trajectory between malignant, non-malignant and metastasis cells. We also identified four immune cell types including T cells, B cells, dendritic cells and NKT cells. Our results found that the number of T cells and B cells had significantly reduced in CA and PCA tissue compared with metastatic lymph node tissue. Immunostaining with antibodies in tumour tissue was also used to evaluate the existence of cell populations from tumour-infiltrating, which indicated statistical relevance with genetic expression in most tumour samples. The findings of additional marker staining for cells were consistent with the results from single-cell RNA-seq, verifying the authenticity of genetic expression map for specifying cell types. Tumours are considered to escape the monitoring of natural immunity either by decrease of the immune systems response or by activation of immunosuppression. The decrease of T cells and B cells in CA and PCA tissue may be a critical determining factor through which the immunosuppression pathway operates, however the immune system response is normal in metastatic lymph node tissue. Our results may provide a therapeutic strategy of OSCC by overcoming the exhaustion of T cells or B cells and searching for new immune checkpoint targets, which have been reported to be significantly effective in the treatment of some cancers^21^. It is interesting to note that the number of NKT cells has significantly increased in CA and PCA tissue when compared with metastatic lymph node tissue, which suggested that NKT cells were activated in the OSCC state.

And beyond that, our data reveals a significant step towards the comprehensive understanding of intra-tumoral expression heterogeneity in SPOSCC after radiotherapy of NPC, which contributes to distinguishing the therapy between primary OSCC and SPOSCC after radiotherapy of NPC. Subsequent cell populations have been verified based on known surface marker expression. Our results found that the number of T cells and neutrophils has significantly reduced in SPOSCC after radiotherapy of NPC compared with CA, PCA and metastatic lymph node tissue. T cells on account of a high loss feature have become targets of immune checkpoint blockade^7^. Our finding can provide a better therapeutic strategy on SPOSCC after radiotherapy of NPC by finding blocking antibodies of T cells or neutrophils to overcome T-cell or neutrophils exhaustion.

In our single-cell data set, the results found that CD1C-CD141-dendritic cells and Natural killer T (NKT) cells were remarkably expressed in CA group. C15orf48, as a novel gene, was significantly higher in CA than that in PCA and LNM, and was highly expressed in CD1C-CD141-dendritic cells and Natural killer T (NKT) cells. The C15orf48 gene was recently identified as an inflammatory response related gene, which is expressed in many organs of the human body. The function of C15orf48 is unknown, but it may have some functions in the occurrence and development of squamous cell carcinoma^22^, ^23^. Under-expression of C15orf48 and its aberrant methylation have been demonstrated in squamous cell carcinomas^24^. It was also identified as a potential tumour prognostic biomarker and immunotherapy target of other cancers^24^-^27^. In this work, among immune cell infiltrations mostly obtained from OSCC, a significant increase in the number of immune cells-CD1C-CD141-dendritic cells and NKT cells with high inflammatory response gene expression was found, which implied an inflammatory reaction in progress. Most CD1C-CD141-dendritic cells and NKT cells expressed C15orf48, which suggested that C15orf48 was a potential target of checkpoint inhibition for future new drugs. Interestingly, these T cells also manifested immuno-suppressive phenotypes of exhaustion. Our results found that IL-1 receptor antagonist (IL1RN) was significantly higher in OSCC than that in PCA, LNM and was only highly expressed in basal cells and epithelial cells. IL1RN belongs to the interleukin-1 (IL-1) family of cytokines, and is the most effective molecule in the innate immune system^28^, ^29^. IL1RN was firstly discovered as a natural antagonist of IL-1 that inhibits IL-1 signaling^30^. Once the balance between IL1RN and IL-1 is disturbed, physiological disorders of the human body would lead to the higher incidence of many cancers. Previous studies have reported that IL1RN was a biomarker of many cancers^31^-^33^, including the biological function of IL1RN in OSCC. Previous studies have also suggested that IL1RN could represent a reliable biomarker for the early diagnosis of OSCC[34-36]and low expression of IL1RN predicted poor overall survival in OSCC patients^37^. Additional IL1RA intracellular isoforms have been found in epithelial cells. Here we also show that high expression of IL1RA is found in epithelial cells^38^. Both existing literature and our results suggest that Targeting IL1RN in epithelial cells might provide novel orientations in curing OSCC.

In summary, these findings proved the scope and potential influence of heterogeneity within the tumour. Moreover, single-cell transcriptome profiling could authenticate and characterize crucial subpopulations in clinical to develop more effective targeted therapies of OSCC. Further, these findings in our search implied the requirement for single-cell genetic expression profiling projects on a small scale for comprehensively characterizing of heterogeneous tumours such as SPOSCC after radiotherapy of NPC.

## Figures and Tables

**Table 1:** Clinical characteristics of patients in immunohistochemical verification

Variable	CA	PCA	LNM	SPOSCC after radiotherapy of NPC	p value
Total patients	41	51	67	11	
					
Age range	23-79	23-79	23-79	42-67	0.574
Mean ± standard	53.22±12.97	55.53±12.64	53.87±13.45	58.55±7.8	
≤50 years/>50 years	14/27	17/34	25/42	2/9	
					
Gender (Male/Female)	29/12	36/15	49/18	6/5	0.665
					
Tumor stage(I-II/III-IV)	24/17	35/16	44/23	7/4	0.787
					
Tumor size (<3cm/≥3cm)	17/24	20/31	33/34	7/4	0.404
					
Differentiated degree (HD/LD)	28/13	37/14	46/21	7/4	0.929
					
Smoking (Smoking/No smoking)	16/25	13/38	21/46	1/10	0.218
					
Drinking (Drinking/No drinking)	9/32	10/41	14/53	2/9	0.989

CA: Carcinoma; PCA: Para-carcinoma; LNM: lymph node metastasis; SPOSCC: Second Primary Oral Squamous Cancer; NPC: nasopharyngeal carcinoma; HD: high differentiation; LD: low differentiation.

**Table 2:** The expression level of biomarkers of cell clusters in tissue

No.	Gene name	Expression level	CA	PCA	LNM	P value (CA/PCA)	P value (CA/LNM)	P value (PCA/LNM)
1	IL1RA	Positive /Negative	23/18	17/34	22/47	0.029	0.013	0.867
2	C15orf48	Positive /Negative	20/21	10/41	16/51	0.003	0.008	0.579
3	CD3	Positive /Negative	10/31	9/42	16/51	0.427	0.952	0.412
4	CD20	Positive /Negative	15/26	11/40	59/8	0.112	0.000	0.000
5	CD79A	Positive /Negative	7/34	12/39	52/15	0.447	0.000	0.000
6	S100A2	Positive /Negative	34/7	27/24	36/31	0.002	0.002	0.932
7	CD31	Positive /Negative	5/36	11/40	58/9	0.238	0.000	0.000
8	CD66	Positive /Negative	36/5	44/7	62/5	0.828	0.410	0.265
9	Cytokeratin 5	Positive /Negative	26/15	28/23	15/52	0.410	0.000	0.000

CA: Carcinoma; PCA: Para-carcinoma; LNM: lymph node metastasis

**Table 3:** The expression level of biomarkers in SPOSCC after radiotherapy of NPC

No.	Gene name	Expression level	SPOSCC	CA	PCA	LNM	P value (/CA)	P value (/PCA)	P value (/LNM)
1	IL1RA	Positive /Negative	10/1	23/18	17/34	22/47	0.033	0.000	0.000
2	C15orf48	Positive /Negative	8/3	20/21	10/41	16/51	0.157	0.000	0.001
3	CD3	Positive /Negative	3/8	10/31	9/42	16/51	0.732	0.464	0.808
4	CD20	Positive /Negative	0/11	15/26	11/40	59/8	0.017	0.089	0.000
5	CD79A	Positive /Negative	1/10	7/34	12/39	52/15	0.515	0.286	0.000
6	S100A2	Positive /Negative	9/2	34/7	27/24	36/31	0.931	0.078	0.081
7	CD31	Positive /Negative	10/1	5/36	11/40	58/9	0.000	0.000	0.690
8	CD66	Positive /Negative	6/5	36/5	44/7	62/5	0.013	0.016	0.000
9	Cytokeratin 5	Positive /Negative	9/2	26/15	28/23	15/52	0.248	0.099	0.000

CA: Carcinoma; CPA: Para-carcinoma; LNM: lymph node metastasis; SPOSCC: Second Primary Oral Squamous Cell Cancer; NPC: nasopharyngeal carcinoma

**Table 4:** Clinicopathologic findings of biomarkers in patients with OSCC

No.	Gene name	Positive number/Negative number			P value
1	IL1RA	Age	≤50 years (8/8)	>50 years (15/10)	0.529
		Gender	Male (15/14)	Female (8/4)	0.38
		Stage	I/II stage (12/12)	III/IV stage (11/6)	0.35
		Tumor size	<3 cm (9/8)	≥ 3 cm (14/10)	0.732
		Differentiation degree	High differentiation (17/11)	Low differentiation (6/7)	0.382
		Smoking state	Smoking (11/5)	No smoking (12/13)	0.192
		Drinking state	Drinking (7/2)	No drinking (16/16)	0.138
					
2	C15orf48	Age	≤50 years (7/7)	>50 years (13/14)	0.91
		Gender	Male (15/14)	Female (5/7)	0.558
		Stage	I/II stage (10/12)	III/IV stage (10/7)	0.408
		Tumor size	<3 cm (10/7)	≥ 3 cm (10/14)	0.279
		Differentiation degree	High differentiation (16/12)	Low differentiation (4/9)	0.116
		Smoking state	Smoking (9/7)	No smoking (11/14)	0.444
		Drinking state	Drinking (4/5)	No drinking (16/16)	0.768
					
3	CD3	Age	≤50 years (4/11)	>50 years (6/21)	0.746
		Gender	Male (9/18)	Female (1/11)	0.099
		Stage	I/II stage (5/19)	III/IV stage (5/12)	0.529
		Tumor size	<3 cm (6/11)	≥ 3 cm (4/20)	0.171
		Differentiation degree	High differentiation (5/23)	Low differentiation (5/8)	0.153
		Smoking state	Smoking (5/11)	No smoking (5/20)	0.413
		Drinking state	Drinking (4/7)	No drinking (6/26)	0.233
					
4	CD20	Age	≤50 years (10/4)	>50 years (5/22)	0.001
		Gender	Male (11/18)	Female (4/8)	0.781
		Stage	I/II stage (12/12)	III/IV stage (3/14)	0.034
		Tumor size	<3 cm (7/10)	≥ 3 cm (8/16)	0.607
		Differentiation degree	High differentiation (8/20)	Low differentiation (7/6)	0.118
		Smoking state	Smoking (10/6)	No smoking (5/20)	0.006
		Drinking state	Drinking (4/5)	No drinking (11/21)	0.58
					
5	CD79A	Age	≤50 years (3/11)	>50 years (4/23)	0.594
		Gender	Male (3/26)	Female (4/8)	0.075
		Stage	I/II stage (3/21)	III/IV stage (4/13)	0.355
		Tumor size	<3 cm (3/14)	≥ 3 cm (4/20)	0.934
		Differentiation degree	High differentiation (2/26)	Low differentiation (5/8)	0.013
		Smoking state	Smoking (2/14)	No smoking (5/20)	0.534
		Drinking state	Drinking (2/7)	No drinking (5/27)	0.642
					
6	S100A2	Age	≤50 years (12/2)	>50 years (22/5)	0.733
		Gender	Male (23/6)	Female (11/1)	0.339
		Stage	I/II stage (22/2)	III/IV stage (12/5)	0.077
		Tumor size	<3 cm (15/2)	≥ 3 cm (19/5)	0.447
		Differentiation degree	High differentiation (23/5)	Low differentiation (11/2)	0.845
		Smoking state	Smoking (14/2)	No smoking (20/5)	0.534
		Drinking state	Drinking (7/2)	No drinking (27/5)	0.642
					
7	CD31	Age	≤50 years (2/12)	>50 years (3/24)	0.768
		Gender	Male (3/26)	Female (2/10)	0.574
		Stage	I/II stage (2/22)	III/IV stage (3/14)	0.369
		Tumor size	<3 cm (4/13)	≥ 3 cm (1/23)	0.062
		Differentiation degree	High differentiation (3/25)	Low differentiation (2/11)	0.671
		Smoking state	Smoking (4/12)	No smoking (1/24)	0.045
		Drinking state	Drinking (4/5)	No drinking (1/31)	0.001
					
8	CD66	Age	≤50 years (12/2)	>50 years (23/4)	0.964
		Gender	Male (25/4)	Female (11/1)	0.627
		Stage	I/II stage (20/4)	III/IV stage (16/1)	0.299
		Tumor size	<3 cm (15/2)	≥ 3 cm (21/3)	0.943
		Differentiation degree	High differentiation (25/3)	Low differentiation (11/2)	0.671
		Smoking state	Smoking (15/1)	No smoking (21/4)	0.352
		Drinking state	Drinking (9/0)	No drinking (27/5)	0.206
					
9	Cytokeratin 5	Age	≤50 years (8/6)	>50 years (18/9)	0.548
		Gender	Male (16/13)	Female (10/2)	0.089
		Stage	I/II stage (18/6)	III/IV stage (8/9)	0.067
		Tumor size	<3 cm (13/4)	≥ 3 cm (13/11)	0.144
		Differentiation degree	High differentiation(20/8)	Low differentiation(6/7)	0.118
		Smoking state	Smoking (13/3)	No smoking (13/12)	0.058
		Drinking state	Drinking (7/2)	No drinking (19/13)	0.311
